# Structural Basis of Inhibition of DCLK1 by Ruxolitinib

**DOI:** 10.3390/ijms22168488

**Published:** 2021-08-06

**Authors:** Dong Man Jang, Hyo Jin Lim, Hyunggu Hahn, Yeon Lee, Hark Kyun Kim, Hyoun Sook Kim

**Affiliations:** Research Institute, National Cancer Center, Goyang 10408, Gyeonggi, Korea; jdm721@ncc.re.kr (D.M.J.); mayhj4717@gmail.com (H.J.L.); hh2763@nyu.edu (H.H.); ylee@ncc.re.kr (Y.L.)

**Keywords:** doublecortin-like kinase 1, DCLK1, kinase inhibitor, anticancer, structure-based drug discovery, ATP-competitive inhibitor

## Abstract

Given the functional attributes of Doublecortin-like kinase 1 (DCLK1) in tumor growth, invasion, metastasis, cell motility, and tumor stemness, it is emerging as a therapeutic target in gastrointestinal cancers. Although a series of specific or nonspecific ATP-competitive inhibitors were identified against DCLK1, different types of scaffolds that can be utilized for the development of highly selective inhibitors or structural understanding of binding specificities of the compounds remain limited. Here, we present our work to repurpose a Janus kinase 1 inhibitor, ruxolitinib as a DCLK1 inhibitor, showing micromolar binding affinity and inhibitory activity. Furthermore, to gain an insight into its interaction mode with DCLK1, a crystal structure of the ruxolitinib-complexed DCLK1 has been determined and analyzed. Ruxolitinib as a nonspecific DCLK1 inhibitor characterized in this work is anticipated to provide a starting point for the structure-guided discovery of selective DCLK1 inhibitors.

## 1. Introduction

Cancer was once considered a disease arising from genetic mutations, but it is now understood that epigenetic changes that elicit the deregulation of signaling pathways are sufficient to cause malignancies [1]. Phosphorylation is one of the most common PTMs involved in the regulation of multiple biological processes and overexpression of kinase. Dysregulation of kinases can lead to aberrant activation of signaling pathways and oncogenesis of multiple types of cancer [2,3].

Doublecortin-like kinase 1 (DCLK) is a microtubule-associated serine/threonine kinase that contains two tandem doublecortin (DCX) domains on its N-terminus [4]. Since doublecortins recognize microtubules, DCLK1 was identified as a microtubule-associated protein (MAP) [5]. The C-terminal kinase domain shows high resemblance to Ca^2+^/calmodulin-dependent protein kinase 1 (CaMKI) domain [6]. Between the domains is a flexible PEST linker region rich in proline, glutamate, serine, and threonine and harboring multiple phosphorylation sites [7]. Although the primary role of DCLK1 is focused on the regulation of cellular signaling pathways, its role has also been established to be involved in neurogenesis and neuronal migration [6], presumably by promoting tubulin polymerization [8]. Especially, recent studies have identified DCLK1 as a cancer stem cell (CSC) marker of malignant cells in the small intestine and pancreas [9,10,11,12,13]. Overexpression of DCLK1 was found essential for cancer progression, epithelial-mesenchymal transition (EMT), and metastasis of many types of cancer through downregulating tumor suppressor microRNAs [14,15,16,17,18]. Suppressing DCLK1 expression or its kinase activity resulted in decreased expression of markers of EMT and pluripotency [19,20]. In addition, high expression of DCLK1 splicing variants that lack the N-terminal DCX and PEST region was identified in human colon adenocarcinomas (hCRCs) and leads to significantly decrease overall survival in a cohort of CRC patients [21,22]. Moreover, cancer-related mutations within the kinase domain were found to adversely affect the kinase activity and/or the structural integrity of DCLK1 [8]. Therefore, regulation of DCLK1 is complicated, but the inhibition of its kinase activity seems to be a promising strategy in cancer treatment and the elimination of CSCs.

There are numerous inhibitors of kinases, developed to regulate crucial signaling pathways governing the onset and progression of cancer [23]. Since the development of a new drug is both time and money consuming, repurposing already approved drugs is gaining much attention [24]. In this respect, we evaluated 2104 clinically applied compounds for DCLK1 binding using the differential scanning fluorimetry method to identify different types of scaffolds that bind and stabilize the kinase domain of DCLK1 proteins. It is followed by the validation of selected compounds by surface plasmon resonance (SPR) and homogeneous time-resolved fluorescence (HTRF) methods, with ruxolitinib being the most potent against DCLK1. To better understand the interaction mode of DCLK1 with ruxolitinib, the crystal structure of DCLK1 in complex with ruxolitinib was investigated. The binding mode of ruxolitinib that is ensconced in the ATP-binding site of DCLK1 rationalizes its potency and serves a foundation for future rational optimization. Accordingly, using biophysical and biochemical screening as well as structural analysis, we have identified a new core structure for the further optimization and the design of selective and therapeutically effective inhibitors against DCLK1.

## 2. Results

### 2.1. Screening with Clinical Library Reveals Small-Molecule Doublecortin-Like Kinase 1 (DCLK1) Binders

**Figure 1:**
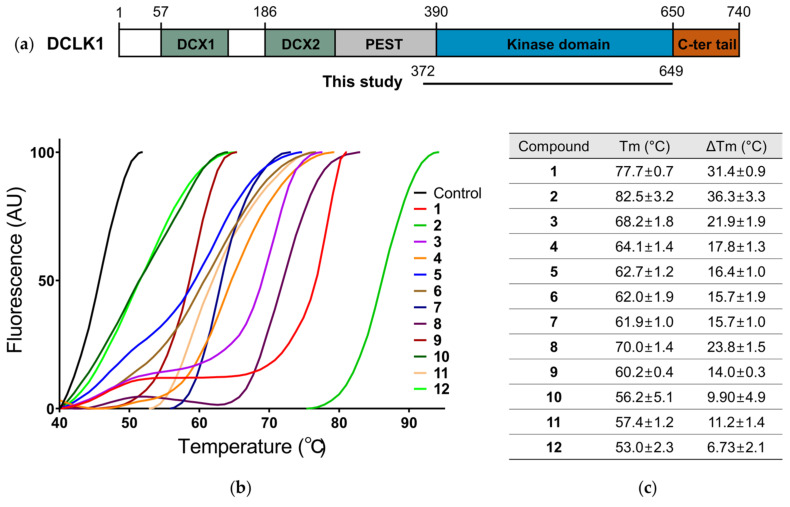
A clinical library screening against the kinase domain of Doublecortin-like kinase 1 (DCLK1_KD_) using a differential scanning fluorimetry assay. (**a**) The schematic diagram of domain organization of the full length DCLK1. (**b**) The thermal melting curves for DCLK1_KD_ proteins upon incubation of compounds are produced using a temperature increment of 0.05 °C from 25 °C to 99 °C with a fluorescent probe. From a clinical library of 2104 compounds, the twelve representative compounds (**1**–**12**) and dimethyl sulfoxide (DMSO) as a control are selected for drawing the thermal melting curves. (**c**) A table for melting temperatures (Tm, °C) and delta melting temperatures (∆Tm, °C) against a control. The data for Tm and ∆Tm are presented as the mean ± standard deviation (SD) of five independent experiments.

Here, we conducted a screening with the clinical library to identify novel chemical entities that bind to the purified DCLK1 kinase domain (DCLK1_KD_; Gly372–Asp649) (Figure 1a), since repurposing or repositioning existing drugs could have potential benefits such as lower overall risk and an accelerated optimization. Using a differential scanning fluorimetry (DSF) assay in a real-time PCR instrument, we screened the clinical library of 2104 compounds (Supplementary Figure S1a) and selected 12 compounds (compounds **1**–**12**) that significantly increase the thermal melting temperature (Tm) shift by more than 4 °C (Figure 1b,c, Table S1). The chemical structures of the compounds are diverse, yet some of them share core structures, such as **1**:**3** and **4**:**7** (Table S1).

### 2.2. Ruxolitinib Shows Significant off Target Activity against DCLK1

**Figure 2:**
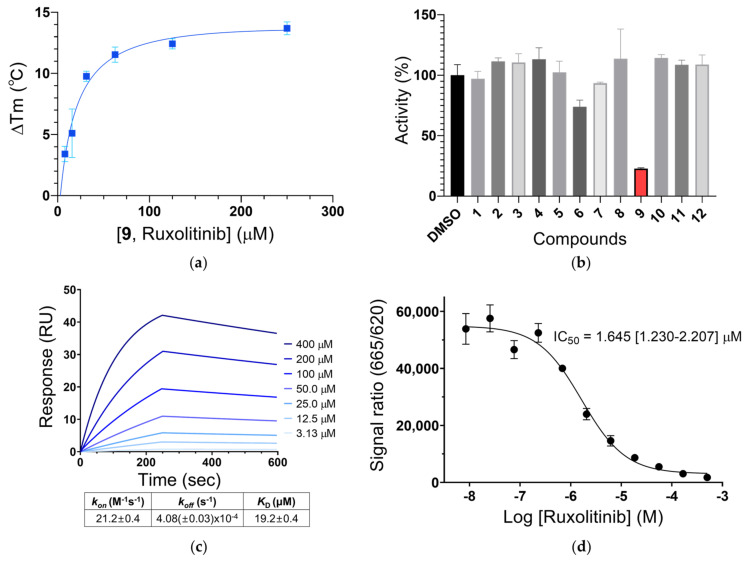
The biochemical properties of the most potent inhibitor ruxolitinib against the DCLK1_KD_. (**a**) Dose-dependent thermal shift of the DCLK1_KD_ by ruxolitinib. Delta Tm (°C) of the DCLK1_KD_ was determined from a DSF assay with and without ruxolitinib (compound **9**) and plotted against the concentration of ruxolitinib from 7.81 to 250 μM. This experiment was performed at least in duplicate for each sample. (**b**) Relative kinase activities of DCLK1_KD_ inhibited by 10 μM compounds. A kinase activity was measured by an end-point inhibition assay using a homogenous time-resolved fluorescence (HTRF) method and normalized toward the respective DMSO activity (100%). (**c**) A surface plasmon resonance (SPR) analysis for a binding affinity between the DCLK1_KD_ and ruxolitinib. SPR sensorgrams show the binding of ruxolitinib at increasing concentrations to immobilized DCLK1_KD_ proteins (colored lines). The values for *K*_D_, *k_on_*, and *k_off_* were calculated from the fitted responses. (**d**) The inhibitory activity of ruxolitinib against DCLK1_KD_ was measured with an HTRF assay. Fluorescence signal ratio at the emission wavelengths of 665 nm and 620 nm in the HTRF assay was plotted against various concentrations of ruxolitinib. An IC_50_ value for DCLK1 was calculated at an ATP concentration of 7.7 μM (*K*_m_). Errors are 95% confidence intervals (CI) for three independent experiments.

When the selected chemicals were validated with various concentrations using DSF assay, only the compound **9**, ruxolitinib (INCB018424) (IUPAC name: (3*R*)-3-cyclopentyl-3-[4-(7*H*-pyrrolo[2,3-d]pyrimidin-4-yl)pyrazol-1-yl]propanenitrile) chemical showed dose-dependent Tm shifts (Figure 2a). In addition, end-point inhibition assays using a homogeneous HTRF method over the 12 compounds revealed that ruxolitinib gives a relatively significant inhibition of DCLK1 (Figure 2b). The direct binding between ruxolitinib and DCLK1 proteins was further confirmed by assessing the binding affinity of ruxolitinib with DCLK1 with the SPR method, giving a dissociation constant (*K*_D_) of 19.2 (±0.4) µM (Figure 2c). At an ATP concentration of 7.7 μM (*K*_m_) (Supplementary Figure S1b), the compound has a half maximal inhibitory concentration (IC_50_) of 1.645 (±0.56) µM against DCLK1 kinase domain (Figure 2d), which is comparable to those from NVP-TAE684 (1.084 µM) and LRRK2-IN-1 (2.346 µM) at 10 µM ATP which were previously reported as DCLK1 inhibitors [8]. However, its activity against DCLK1 is much less potent, when compared with IC_50_ values of ruxolitinib against Janus kinases (JAKs) (3.3 nM for JAK1 and 2.8 nM for JAK2) [25] that are its primary targets, or when compared with that of DCLK1-IN-1 against DCLK1 (57.2 nM at 50 µM ATP) [26] that was developed from the core structure of LRRK2-IN-1 or XMD8-85 as a potent DCLK1 inhibitor. Nonetheless, ruxolitinib exhibits a chemical structure totally different from previously reported DCLK1 inhibitors, which could provide a starting point for the development of effective DCLK1 inhibitors.

### 2.3. Overall Structural Features of the Ruxolitinib-Bound DCLK1

**Figure 3:**
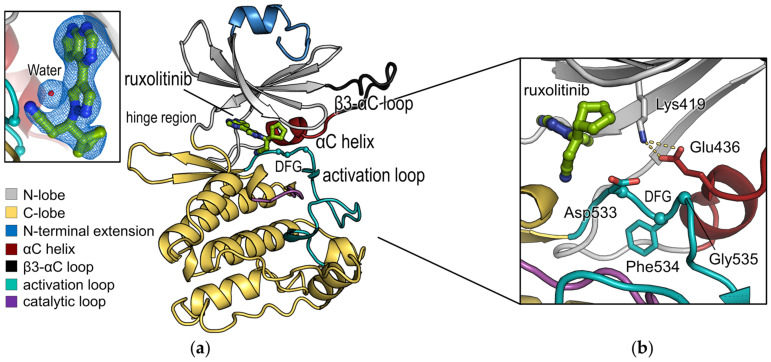
The overall structure of DCLK1_KD·Ruxolitinib_. (**a**) The overall monomer structure is displayed as a cartoon representation colored by domains, as indicated in the colored boxes. Cα atoms for a DFG motif on the activation loop are indicated by spheres. Ruxolitinib and a key water molecule in the binding pocket of the DCLK1_KD_ are shown as green-colored stick and red sphere, respectively. The omit *mFo*-*DFc* maps (contoured at 2.0 σ) for ruxolitinib and the key water molecule are displayed as a blue-colored mesh representation in the inset. (**b**) Close-up view displaying a DFG-in conformation. Ruxolitinib, a DFG motif and surrounding residues are shown as stick representations colored by domains. Cα atoms of DFG motif are indicated with spheres.

To provide reliable structural information for rational design of inhibitors and to further explore the mechanism of action of ruxolitinib, the crystal structure of the DCLK1 kinase domain in the complex of ruxolitinib (DCLK1_KD·Ruxolitinib_) was determined at 2.1 Å resolution by soaking a threefold molar excess of ruxolitinib into the AMPPNP-complexed DCLK1 crystal for substitution. It implies that, to an extent, ruxolitinib outcompetes AMPPNP in the DCLK1 crystal structure via a comparable affinity. In the DCLK1_KD·Ruxolitinib_ structure, we could successfully resolve the structural model for residues 376‒648 in two molecules per an asymmetric unit (ASU), giving *R_work_* and *R_free_* values of 21.7% and 25.1%, respectively (Table 1). The mutual arrangement of the DCLK1 molecules was virtually identical. Expectedly, the structure adopts the overall kinase domain architecture, with an N-lobe and a C-lobe (Figure 3a), where the inter-lobe cleft represents the ATP binding site, which is occupied by the small molecule inhibitor, ruxolitinib (the inset of Figure 3a).

In accordance with the previously reported DCLK1 kinase domain structures [8], the DCLK1_KD·Ruxolitinib_ structure displays an active-state conformation with a DFG-in conformation where the side chain of Asp533 is positioned toward the ATP-binding site and the Phe534 residue is in contact with the αC-helix of the N-terminal lobe (Figure 3b). In addition, an inward disposition of the αC-helix positions Glu436 on the helix to form a salt bridge with strictly conserved Lys419 (Figure 3b).

### 2.4. Dimerization of the Non-Phosphorylated DCLK1

**Figure 4:**
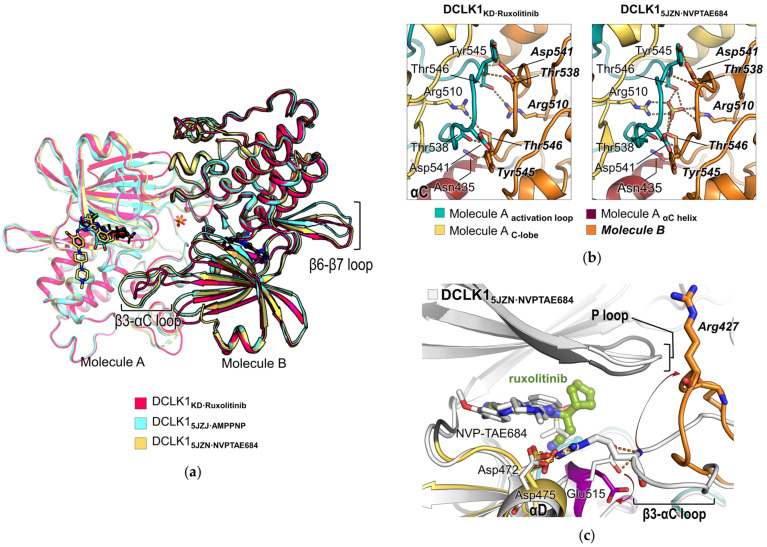
Structural comparison of DCLK1_KD·Ruxolitinib_, DCLK1_5JZN·NVPTAE684_, and DCLK1_5JZJ·AMPPNP_. (**a**) Superimposed view of the dimer structures of the DCLK1_KD_ in complex with ruxolitinib (this study), AMPPNP (PDB ID: 5JZJ), and NVPTAE684 (PDB ID: 5JZN). The overall structures and ligands are shown as cartoon and stick representations, respectively, and colored as indicated in the colored boxes. Monomer structures corresponding to molecule A are shown with a slight transparency on cartoon representations. (**b**) Close-up view of the dimer interface of DCLK1_KD·Ruxolitinib_ and DCLK1_5JZN·NVPTAE684_ structures. Sidechains of residues involved in the interaction between two monomers and the sulfate molecule in the DCLK1_5JZN·NVPTAE684_ structure are shown as stick representations. Hydrogen bonds are indicated by a red dotted line. The activation loop, C-lobe, and αC helix of molecule A and molecule B are colored in cyan, yellow, wine, and orange, respectively, as indicated below. The names of residues from molecule B are labeled as bold italic letters. (**c**) Superimposed view of the active site of DCLK1_KD·Ruxolitinib_ and DCLK1_5JZN·NVPTAE684_ structures. Sidechains of residues in the active site and inhibitors are shown as stick representations. The structure of DCLK1_5JZN·NVPTAE684_ is colored in white. All oxygen and nitrogen atoms on stick representations are colored in red and blue, respectively.

Two DCLK1 molecules in the ASU form a dimeric unit (Figure 4a), showing the surface area buried between them of 1457 Å^2^ per monomer as calculated by the PISA server [27]. The dimer arrangement was shown to be similar with the head-to-tail and face-to-face orientations from the previously reported DCLK1 structures in complex with AMP-PNP or NVP-TAE684 (DCLK1_5JZJ·AMPPNP_ or DCLK1_5JZN·NVPTAE684_, respectively), where the dimerization is stabilized by tight interactions of Thr546 on the activation loop (residues 533‒557) and Arg510 within the catalytic loop HRD motif with a sulfate molecule that is reminiscent of the phosphorylated form of Thr546, thereby allowing an active conformation [8] (Figure 4b, right). However, the DCLK1_KD·Ruxolitinib_ structure shows dimer formation not mediated by any ligand mimicking the phosphorylated form, but still retains active conformation (Figure 4b, left). Arg510^N^^η^ and Thr546^Oγ^ each from different monomeric subunits in the oligomer directly make hydrogen bonds with the distance of 3.6 Å, which is closer than the distance (4.2 Å) between them in DCLK1_5JZN·NVPTAE684_. Moreover, Thr538, Asp541, and Tyr545 located within the activation loop, and Asn435 on the αC helix establish an extensive network of hydrogen bonds and hydrophilic inter-molecular interactions between two activation loops or between the activation loop and the αC helix, meaning that the phosphorylation of the activation loop of DCLK1 might not be prerequisite for maintaining the integrity of the dimeric form.

Instead, more adjacent Arg510 and Thr546 residues appear to make two C-lobes tighten, thereby affecting the orientation of N-lobes. When superposing the dimeric forms of DCLK1_KD·Ruxolitinib_, DCLK1_5JZJ·AMPPNP_, and DCLK1_5JZN·NVPTAE684_, the N-lobe part in DCLK1_KD·Ruxolitinib_ is shown to be certainly rearranged (Figure 4a). A major difference among the superposed dimer structures arises from their loops before αC helices (β3-αC loop) (Figure 4a,c). Their αC helices per se display similar swung-in conformation as a short length (Asn435–Arg442), whereas conformations of β3-αC loops vary depending on whether or not a sulfate is involved in dimerization. In the dimer structure of DCLK1_5JZN·NVPTAE684_ or DCLK1_5JZJ·AMPPNP_, the β3-αC loop of DCLK1-B is stretched toward the active site of DCLK1-A, where the side chain of Arg427 located at the turn of the β3-αC loop interacts with Asp472 and Asp475 of the αD helix and its main chain is further stabilized by hydrogen bonds of Glu515^Oε1,Oε2^, which together constitute the ATP-binding pocket (Figure 4c and Supplementary Figure S2b,c). In contrast, the β3-αC loop of the dimer structure of DCLK1_KD·Ruxolitinib_ is flipped over by the large movement of Arg427^Nη^ (18.4 Å), resulting in the concomitant shift of Glu515^Oε^ (4.9 Å) (Figure 4c and Supplementary Figure S2a,c). These structural rearrangements alter the overall shape of the active-site cavity as shown in Supplementary Figure S2a,b, which in turn might affect kinase activity, possibly explaining the previous result that the phospho-mimetic T546E mutant displayed slightly elevated activity than the wild-type (WT) protein [8]. Therefore, it is noteworthy that with respect to the structure-based inhibitor design, DCLK1 has considerable flexibility not only in the active site, but also in the cavity formed from dimerization in a face-to-face fashion (Supplementary Figure S2d,e).

### 2.5. Interaction Mode of Ruxolitinib with DCLK1

The compound was shown to be directed at the active-like conformation with a DFG-in conformation with an inward disposition of the αC-helix, which positions Glu436 on the helix to form a salt bridge with Lys419. These elements appear to be a pocket conductive to ruxolitinib binding. Ruxolitinib was modeled into electron density in each protein monomer in ASU of the DCLK1_KD·Ruxolitinib_ structure. The binding states of the two ruxolitinib molecules are similar, but the omit map for the ligand within chain A is clearer than the other. Henceforth, we will focus on the ruxolitinib molecule modeled in chain A.

**Figure 5:**
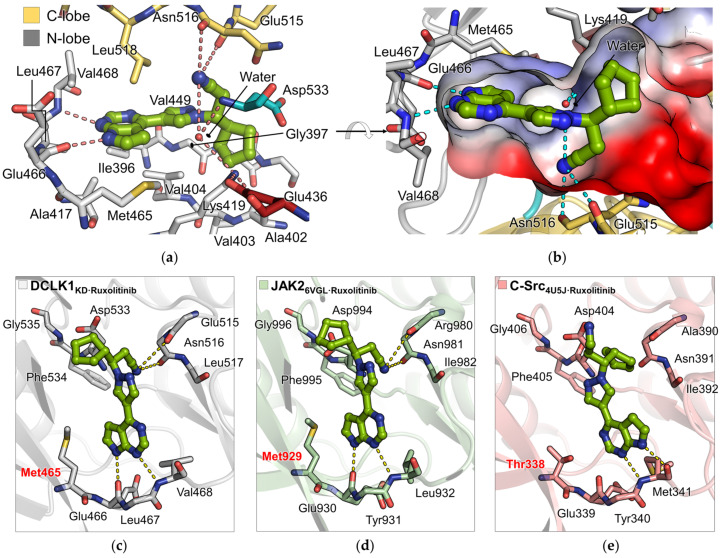
The binding mode of ruxolitinib in the structures of DCLK1, JAK2, and C-Src. (**a**,**b**) Close-up view of ruxolitinib bound to DCLK1_KD·Ruxolitinib_. (**a**) Ruxolitinib and surrounding residues on N-lobe, C-lobe, activation loop, and αC-helix of DCLK1 are shown as stick representations colored in green, grey, yellow, cyan, and wine, respectively. (**b**) The binding pocket for ruxolitinib is depicted by an electrostatic potential surface diagram, where positive and negative charges are colored in blue and red, respectively. (**c**–**e**) Comparison of ruxolitinib bound to the kinase domains of (**c**) DCLK1, (**d**) JAK2 (PDB ID: 6VGL), and (**e**) c-Src (PDB ID: 4U5J). Ruxolitinib and surrounding residues of DCLK1, JAK2, and c-Src are shown as stick representations colored in green, grey, pale green, and salmon, respectively. Gatekeeper residues are labeled in red. All hydrogen bonds are indicated by colored dotted lines. Oxygen, nitrogen, and sulfur atoms are colored in red, blue, and yellow, respectively.

Ruxolitinib is bound to the ATP-binding site between the N- and C-terminal lobes of DCLK1 and surrounded by the hinge region, activation loop, and αC-helix (Figure 3a), which does not affect the oligomeric state of DCLK1_KD_ in solution (Supplementary Figure S3). In details, the pyrrolopyrimidine ring of ruxolitinib is sandwiched between Val404 and Ala417 of N-lobe and Leu518 of C-lobe via hydrophobic contacts and is oriented toward the hinge region to form two hydrogen bonds with the main chains of Glu466 and Val468 with the distances of 2.9 Å and 3.2 Å, respectively (Figure 5a). The nitrile on the propanenitrile group confers a significant affinity for DCLK1, making an intensive network of interactions with the main chains of Glu515 and Asn516 on the C-lobe, which is in a similar position with the interactions for the ribose sugar of AMPPNP in the DCLK1_5JZJ·AMPPNP_ structure and with the side chains of invariant Lys419 and Glu436 on the N-lobe via a water molecule (Figure 5a). The cyclopentyl group of ruxolitinib contributes to the tight binding by occupying a hydrophobic groove underneath a conserved glycine-rich loop, or P-loop, which connects the β1 and β2 strands (Figure 5b and Supplementary Figure S4).

Ruxolitinib has been reported to inhibit JAK kinases to a much higher extent than Src kinases [28]. In order to expand the structural characteristics of ruxolitinib bound to different kinases related with its potency, we compared the binding mode of ruxolitinib in the DCLK1 structure with those in JAK2 and c-Src structures published so far (Figure 5c–e). Interestingly, the binding mode of ruxolitinib in the structure of DCLK1 resembles to that in the JAK2 structure with respect to the direction of ruxolitinib in the binding pocket of kinases, where the pyrrolopyrimidine ring of ruxolitinib in the DCLK1 and JAK2 structures makes two hydrogen bonds with the hinge region and the pyrrole groups were oriented toward Met465 and Met929, respectively, known as a gatekeeper (Figure 5c,d). In contrast, ruxolitinib in the structure of c-Src is flipped such that the pyrrole group remains pointed in the opposite direction from the gatekeeper residue, Thr338 in c-Src, while the pyrrolopyrimidine ring still forms two hydrogen bonds with the hinge region (Figure 5c,e). Upon the flipped direction of ruxolitinib, the propanenitrile group in both structures of DCLK1 and JAK2 interacts with the main chains of residues 515–516 (residues 980–981 for JAK2) on the αE-β6 loop of C-lobe via two hydrogen bonds unlike that of c-Src (Figure 5c–e). Considering the higher binding affinity of ruxolitinib for JAK kinases than c-Src kinases, the similar binding mode of ruxolitinib bound to DCLK1 and JAK2 suggests that DCLK1 harbors a considerably favorable binding pocket for ruxolitinib.

### 2.6. Comparison of the Binding Mode of Ruxolitinib with Other DCLK1 Inhibitors and ATP

**Figure 6:**
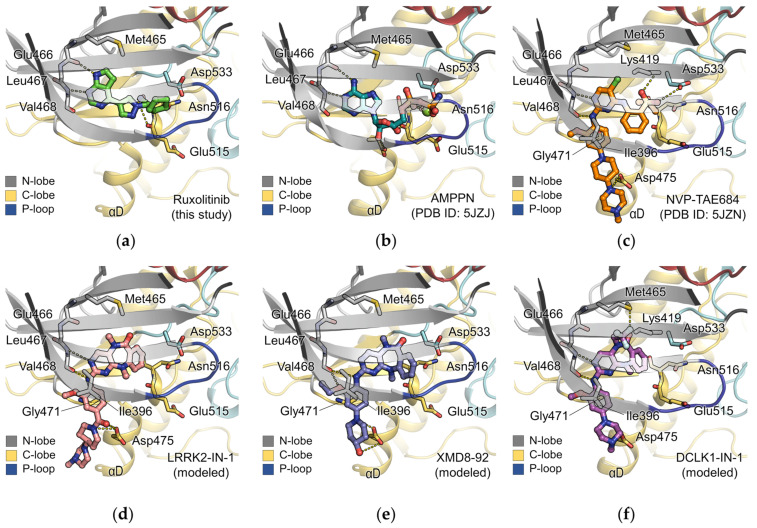
The binding mode comparison of DCLK1 inhibitors. (**a**–**c**) Binding modes of inhibitors and AMPPN in the structures of DCLK1_KD·Ruxolitinib_, DCLK1_5JZJ·AMPPNP_ (PDB ID: 5JZJ), and DCLK1_5JZN·NVPTAE684_ (PDB ID: 5JZN). (**d**–**f**) Binding modes of inhibitors modeled by docking to the DCLK1_KD_ structures via AutoDock Vina program (The Scripps Research Institute, CA, USA). The coordinates for LRRK2-IN-1 and XMD8-92 and the coordinate for DCLK1-IN-1 are docked to the ligand-free structure of DCLK1_KD·Ruxolitinib_ and DCLK1_5JZN·NVPTAE684_, respectively. Ruxolitinib, AMPPN, NVP-TAE684, LRRK2-IN-1, XMD8-92, and DCLK1-IN-1 are shown as ball-and-stick representations colored in green, teal, orange, pink, salmon, slate, and magenta, respectively. Interacting residues with inhibitors are shown as stick representations colored by domains. All oxygen and nitrogen atoms are colored in red and blue, respectively. Fluorine atoms of DCLK1-IN-1 are colored in light blue.

Although the binding mode of ruxolitinib for DCLK1 resembles that for the primary target kinase JAK2, the binding affinity and efficacy of ruxolitinib against DCLK1 is much lower. To extend the knowledge for more potent and effective inhibitors against DCLK1, we compared the structure of ruxolitinib bound to DCLK1 with ATP or other DCLK1 inhibitors, including NVP-TAE684, LRRK2-IN-1, XMD8-92, and DCLK1-IN-1 using their structures bound to DCLK1 or molecular docking models. In the ATP binding site of DCLK1, the inhibitors, as well as ATP, are using nitrogen atoms to interact with main chains of the hinge region in DCLK1 (Figure 6a–f). However, ruxolitinib is composed of a completely different scaffold compared to the other inhibitors, which results in a distinct binding mode (Figure 6a). In particular, the hydrogen bonding network between the propanenitrile group of ruxolitinib and the main chains of residues 515‒516 is absent in the other inhibitors. In addition, the superposition of ruxolitinib-, AMPPN-, and NVP-TAE684- bound DCLK1 structures shows that the cyclopentyl group of ruxolitinib induces the upward movement of the glycine rich P-loop via hydrophobic contacts (Supplementary Figure S4). On the other hand, the sulfonyl group of NVP-TAE or the phosphate group of AMPPN exhibits polar interactions with side chains of Lys419 and Asp533 (Figure 6b,c).

The most noticeable difference between ruxolitinib and other DCLK1 inhibitors is that it makes physical contacts with the helix αD (Figure 6a–f). Due to the large size of the other DCLK1 inhibitors spanning the molecular weight of 472–612 Da compared to ruxolitinib of 306 Da, the inhibitors harbor additional moieties that are stretching out toward the C-lobe and forming polar interactions with the helix αD (Figure 6c–f). Indeed, the inhibitors LRRK2-IN-1, XMD8-92, and DCLK1-IN-1 have been developed from the same scaffold and are shown to exhibit similar binding modes by having a polar interaction network with Asp475 on helix αD and favorable van der Waals interactions sandwiched between Ile396 on the β1 strand and Gly471 on the hinge region (Figure 6d–f). Among them, DCLK1-IN-1, developed as a potent and selective inhibitor of DCLK1, was substituted with a trifluoroethyl group in the diazipinone amide, creating a putative fluorine-sulfur contact with the gatekeeper Met465 (Figure 6f). Taken all together, the newly discovered interaction mode from the ruxolitinib-bound DCLK1 structure and the re-interpretation regarding the binding modes of the known inhibitors against DCLK1 will provide a valuable and comprehensive information for the further optimization and development of a promising and novel DCLK1 inhibitor.

## 3. Discussion

Even though DCLK1 has emerged as a cancer stem cell marker candidate for pancreatic cancer, intestinal cancer, and colorectal cancer, the structural knowledge of the putative inhibitors with various types of scaffolds targeting the DCLK1 kinase domain remains currently limited. In this respect, we identified several scaffolds that bind and stabilize the DCLK1 kinase domain from 2104 clinically applied compounds using a DSF method (Table S1) and validated selected compounds in binding affinity and inhibitory activity via SPR and HTRF methods. Then, we report a complex structure of DCLK1 kinase domain with the most potent inhibitor ruxolitinib, which has been originally developed as a Janus kinases (JAK1 and JAK2) inhibitor for treatment of myelofibrosis [29]. Ruxolitinib in the structure of DCLK1 kinase domain exerts as an ATP-competitive inhibitor and adopts a similar binding mode in JAK2 more than in c-Src through its propanenitrile group in the interaction with the C-lobe of kinases. In addition, the structural comparison of ruxolitinib and other DCLK1 inhibitors provides an insight about a novel scaffold against the DCLK1 kinase domain, which can be further exploited to design more selective inhibitors.

We identified ruxolitinib for targeting the kinase activity of DCLK1 from thousands of clinical compounds. Drug repurposing is an attractive approach of drug discovery to minimize the risks and costs that come from the development of new drugs and occasionally harnesses unintended effects of original drugs, so called off-target effects, in new therapeutic opportunities [30]. In particular, kinase inhibitors occupy the majority of off-target based drug repurposing cases in cancer chemotherapy since (1) kinase inhibitors tend to be notoriously unselective and (2) the dysregulation of many kinases is involved in driving cancer [30]. For example, tyrosine kinase inhibitor crizotinib was originally designed to inhibit c-MET involved in tumor proliferation and metastasis but is also used as an anti-cancer medication for the treatment of non-small cell lung carcinoma (NSCLC) by targeting echinoderm microtubule-associated protein-like 4 (EML4) fused anaplastic lymphoma kinase (ALK) [31,32]. Ruxolitinib is widely indicated for the treatment of the myeloproliferative neoplasms (MPNs) via inhibition of dysregulated janus kinases (JAK1 and JAK2) despite the side effects such as thrombocytopenia, anemia, and neutropenia [25]. However, ruxolitinib also exhibited off-target effects by inhibiting various kinases at the concentration of 1 μM, among which the inhibition for ROCK was suggested to impair the migration of dendritic cells [33,34]. In this regard, our results added a novel off-target inhibitory effect of ruxolitinib against the kinase activity of DCLK1 and proved its ATP competitive inhibition through the complex structure. Moreover, the binding mode of ruxolitinib in the DCLK1 kinase domain is almost identical to that in JAK2, the original target protein of ruxolitinib, which verifies the off-target inhibition of ruxolitinib for DCLK1. Thrombocytopenia, which is mentioned as an adverse effect of ruxolitinib, has also been controversial as having a negative regulatory role of JAK2 in thrombopoiesis at the end of megakaryocyte differentiation was suggested [35]. Considering a possible action of ruxolitinib toward other kinases such as DCLK1, the discovery and further studies of the novel off-target effect may enable the off-target-based drug repurposing of ruxolitinib.

Nevertheless, the binding affinity (*K*_D_ = 19.2 µM) and inhibitory activity (IC_50_ = 1.6 µM) of ruxolitinib for DCLK1 seems to be too weak to elicit a dominant biological response through specifically targeting the DCLK1 kinase domain. Among kinases, the binding mode of ruxolitinib has been elucidated for JAK2, c-Src, and DCLK1 to date. As an original target protein, JAK2 exhibited potent inhibitory activity for ruxolitinib with an IC_50_ value of 2.8 nM (at 1 mM ATP), whereas c-Src and DCLK1 kinases had micromolar IC_50_ values [25,28]. Given that a kinase inhibitor with an ATP-competitive mode is highly affected by a gatekeeper residue [36], we had posited a more similar binding affinity of ruxolitinib against JAK2 and DCLK1 as they contained a conserved methionine residue as a gatekeeper than that against c-Src with a threonine residue. Even though residues of DCLK1 and JAK2 kinases participating in the interaction with ruxolitinib were almost identical, about 25-fold stronger binding affinity of ruxolitinib for JAK2 was measured in vitro assay (*K*_D_ = 804 nM, measured by microscale thermophoresis) [37]. In order to develop a potent inhibitor for DCLK1 with higher affinity, the factors that determine the difference of binding affinities for ruxolitinib between DCLK1 and JAK2 need to be examined. While it was not captured in the crystal structure, our molecular dynamic simulations showed slightly more contacts between ruxolitinib and the hinge region in the JAK2_6VGL·Ruxolitinib_ structure than the DCLK1_KD·Ruxolitinib_ structure (Supplementary Figure S5), which might be a hint to design further inhibitors for DCLK1. Nevertheless, the 25-fold difference of binding affinities for ruxolitinib between DCLK1 and JAK2 cannot be completely addressed with the number of contacts. Therefore, it also implies that ruxolitinib needs an allosteric modulation for its specific binding along with the canonical pocket formed between the N-lobe and C-lobe, which should be further studied for the development of potent inhibitors.

The inhibitors that are selective for the DCLK1 kinase domain have been optimized from the same scaffold containing [1,4]-benzodiazepin-6-one, which are utilized to target various kinases such as tyrosine kinase non receptor 2 (TNK2), leucine-rich repeat kinase 2 (LRRK2), and extracellular signal-regulated kinase 5 (ERK5) [38]. In order to improve the selectivity for DCLK1, the inhibitors have been attached to functional groups that protrude toward the C-lobe and make interactions with the αD helix of the DCLK1 kinase domain. In addition, the compound DCLK1-IN-1 showed successful selectivity for DCLK1 and DCLK2 over 468 kinases by adding a trifluoroethyl group on the [1,4]-benzodiazepin-6-one scaffold [38], which made a hydrophobic contact with the gatekeeper Met465 in our docking model (Figure 6f). However, the [1,4]-benzodiazepin-6-one scaffold is also known to inhibit the bromodomain of bromodomain-containing protein 4 (BRD4), which hampers a precision therapeutic usage due to the pleiotropic role of BRD4 in cell cycle regulation, chromatin organization, and transcriptional regulation [39]. Since the scaffold shown in ruxolitinib has not been revealed in association with the bromodomain of BRD4, it might be a substituent candidate against the DCLK1 kinase domain by excluding a pleiotropic effect through the inhibition of BRD4. Collectively, the development of new inhibitors selective and potent for DCLK1 could be achieved by combining the novel scaffold of ruxolitinib as a DCLK1 inhibitor with the optimized functional groups interacting with the αD helix and gatekeeper Met465 of DCLK. This possible approach leading to a new class inhibitor for DCLK1 can be further explored in the future work.

## 4. Materials and Methods

### 4.1. Cloning, Protein Expression, and Purification

The *DCLK1* gene was PCR-amplified and cloned into the modified pGEX4T1 vector using *NcoI* and *XhoI* restriction enzymes. The resulting recombinant DCLK1 encompassing residues Gly372–Asp649 is fused with an N-terminal His_6_ tag followed by a TEV cleavage site. It was overexpressed in *Escherichia coli* Rosetta2(DE3)pLysS strain. DCLK1 was induced with 0.5 mM isopropyl 1-thio-β-D-galactopyranoside at 18 °C for 22 h using Lysogenic Broth culture. The harvested cell pellet was resuspended in the lysis buffer containing 20 mM Tris-HCl (pH 7.9), 500 mM sodium chloride, 5 mM imidazole, 10% (*w*/*v*) glycerol, and 2 mM β-mercaptoethanol, before disruption by microfluidizer. The cell lysate was centrifuged at 35,000× *g* for 60 min at 4 °C, then filtered through 0.45 μm filter to remove cell debris and any aggregated proteins. The filtered supernatant was loaded onto an Ni-NTA resin (QIAGEN, Hilden, Germany), which was previously equilibrated with the lysis buffer, and DCLK1 was eluted at 60–200 mM imidazole. The eluent was diluted 7-fold with a buffer containing 20 mM Tris-HCl (pH 7.9) before overnight incubation with His_6_-tagged TEV protease. The cleaved protein samples were further purified by size-exclusion chromatography with HiLoad 16/600 Superdex 75 prep grade column (GE Healthcare, Chicago, IL, USA), which was pre-equilibrated with a buffer containing 20 mM Tris-HCl (pH 7.5), 200 mM sodium chloride, 5% (*w*/*v*) glycerol, and 0.5 mM tris(2-carboxyethyl)phosphine (TCEP). The purified DCLK1 harboring the kinase domain (DCLK1_KD_) were concentrated to 8 mg mL^−1^ for crystallization. The purification steps for chemical screening and the kinase assay were as above, except the His_6_-tag cleavage step.

### 4.2. Differential Scanning Fluorimetry (DSF) Assay

We screened the DCLK1 inhibitors with a clinical library of 2104 compounds (provided by Korean Chemical Bank) using the differential scanning fluorimetry (DSF) method. For DSF assay, we used the Applied Biosystems QuantStudio™ 7 Flex System (Thermo Fisher Scientific, Waltham, MA, USA). Melting curves were determined in 384 well plates using a melt curve temperature increment of 0.05 °C from 25 °C to 99 °C in the continuous option. Protein Thermal Shift™ dye kit (Thermo Fisher Scientific) was used to label the DCLK1 protein at a 1-fold final dilution (from 1000× stock solution). Ligand concentrations were tested at about 55-fold excess (250 μM) to the protein (4.57 μM) in 5% final DMSO concentration. Later dose-response thermal shift validation included ligand concentrations from 7.81 to 250 μM. The buffer solution was 20 mM Tris-HCl at pH 7.96, 200 mM sodium chloride, 5% (*v*/*v*) glycerol, and 0.5 mM TCEP. Twenty microliters of each sample were loaded in a 384-well plate, sealed, and centrifuged. Each reaction was run in duplicate or triplicate and repeated on at least two different plates. DCLK1 alone and the negative control containing only 5% DMSO with DCLK1 were included in each plate to serve as a quality control check from batch to batch of protein. The ΔTm was determined from the first derivative of the melt curve with and without a ligand from the library, using the Protein Thermal Shift™ software version 1.3 (Thermal Fisher Scientific, Waltham, MA, USA).

### 4.3. HTRF Assay

The inhibitory activity of selected ligands against DCLK1_KD_ was measured and verified with an HTRF assay. HTRF reagents and buffers in the HTRF KinEASE-STK S1 kit were purchased from Cisbio Bioassays (Cisbio, Bedford, MA, USA), and the assay was performed according to the manufacturer’s instructions. For the end-point inhibition assay, HTRF assays were performed at the concentrations of 10 μM inhibitors with a final DMSO concentration of 5%, 20 μM ATP, 1 μM substrate-biotin, and 1.42 nM DCLK1 (closed to EC_80_ for the enzyme). To draw and fit the Michaelis–Menten curves, HTRF assays at various concentrations of enzyme, substrate, and ATP were performed for the titration IC_50_ values were obtained at 7.70 µM ATP (close to *K*_m_ value for ATP from in vitro assay), 1 μM substrate-biotin, 1.42 nM DCLK1 (closed to EC_80_ for the enzyme), and various concentrations of ruxolitinib. All HTRF assays were implemented in the supplied buffer supplemented with 1 mM DTT, 2 mM MgCl_2_, and 5% DMSO due to the solubility of selected ligands. Fluorescence signals were measured at two wavelengths for the HTRF assay. The excitation wavelength was 320 nm, and the emission wavelengths were 620 and 665 nm. Fluorescence was detected using an Infinite F200pro (Tecan, Männedorf, Switzerland) with a lag time of 150 μs and integration time of 500 μs. Data reduction was carried out following the manufacturer’s guidelines (Ratio = 10,000 × signal 665 nm/signal 620 nm).

### 4.4. Surface Plasmone Resonance (SPR) Analysis of DCLK1

The kinetics and affinity of DCLK1_KD_ with ruxolitinib (CSNpharm, Arlington Heights, IL, USA) were assessed using a Reichert SR7500 SPR dual channel instrument (Reichert, Depew, NY, USA). The DCLK1_KD_ proteins purified in a buffer containing 20 mM HEPES (pH 7.5), 200 mM sodium chloride, and 2 mM TCEP were immobilized using the standard amino coupling at 20 μL min^−1^ on a carboxymethyl dextran hydrogel surface sensor chip (Reichert) until saturation was achieved. The running buffer used in all SPR experiments was 20 mM HEPES at pH 7.5, 200 mM sodium chloride, 2 mM TCEP, and 5% (*v*/*v*) DMSO. All SPR experiments were performed at 25 °C. Seven concentrations (3.13, 12.5, 25.0, 50.0, 100, 200, and 400 μM) of ruxolitinib were prepared in the running buffer. Serial diluted ruxolitinib samples were injected over the DCLK1_KD_-chip at 30 μL min^−1^ for 4 min for association analyses. Subsequently, the running buffer was applied over the chip for additional 6 min (30 μL min^−1^) for dissociation analyses. Regeneration of the chip was carried out using 50 mM sodium hydroxide. Binding was detected as a change in the refractive index at the surface of the chip as measured by the response unit (RU). A reference flow cell was used to record the response by bovine serum albumin (BSA) as a positive control, and the response by BSA was subtracted from each sample. SPR data were fit using the Scrubber2 software [40].

### 4.5. Crystallization, Data Collection, and Structure Determination

Crystals of the DCLK1_KD_ were obtained at 23 °C by the sitting-drop vapor diffusion method. To obtain a diffraction-quality crystal, the DCLK1 samples were pre-incubated with 1 mM AMP-PNP for 30 min at 4 °C. Each sitting drop, prepared by mixing equal volumes (0.3 μL) of the purified protein and a reservoir solution [25% (*w*/*v*) polyethylene glycol (PEG) 3350, 0.1 M Bis-Tris (pH 6.5), and 200 mM magnesium chloride], was equilibrated against 50 μL of the reservoir solution. Subsequently, crystals co-crystallized with AMP-PNP were soaked overnight in the reservoir solution containing 3 mM ruxolitinib and 5% DMSO, and then were moved into a cryoprotectant solution composed of 18.8% (*w*/*v*) PEG 3350, 75 mM Bis-Tris (pH 6.5), 150 mM magnesium chloride, 15 mM ruxolitinib, 5% DMSO, and 25% (*w*/*v*) glycerol for further incubation for 3 h at room temperature. The crystals were flash cooled in a nitrogen gas stream at 100 K and diffraction data were collected up to resolution of 2.1 Å (Table 1). Raw X-ray diffraction data were processed and scaled using the XDS program package [41]. The crystal structure of DCLK1_KD_ was solved by the molecular replacement method with the MolRep program [42], with a protein-only version of Protein Data Bank (PDB) entry 5JZJ [8] as a search model. The initial solution was refined using Refmac5 in the CCP4 suite [43] and the resulting *F*_o_-*F*_c_ map showed clear electron density for ruxolitinib. The model was further improved using iterative cycles of manual model building in COOT [44] and refinement using Phenix.refine [45]. The reliability of refined structural models was evaluated using MolProbity [46] and the Research Collaboratory for Structural Bioinformatics (RCSB) PDB Validation server. Statistics for the data collection and refinement are summarized in Table 1.

### 4.6. Molecular Docking Simulation

Docking studies were conducted via AutoDock Vina software (The Scripps Research Institute, La Jolla, CA, USA). Coordinates for ligands (LRRK2-IN-1, XMD8-92, and DCLK1-IN-1) were generated using Ligand Builder in COOT [44] and docked onto the structure of DCLK1_KD·Ruxolitinib_. In order to view the previously described binding mode of DCLK1-IN-1 [26], the coordinate of DCLK1-IN-1 was docked to the structure of DCLK1_5JZN·NVPTAE684_ (PDB ID: 5JZN). All the docking runs were performed after ligands and water molecules were removed from the structures of DCLK1_KD_. The size for the grid box was set to 30 Å × 26 Å × 24 Å, which encompassed most of the entire DCLK1_KD_ structure. Figures for the docking results were drawn using PyMol (DeLano, W. L. *The PyMOL Molecular Graphics System*; DeLano Scientific: Palo Alto, CA, USA).

### 4.7. Molecular Dynamics Simulation

Molecular dynamics (MD) simulations of DCLK1_KD·Ruxolitinib_ and JAK2_6VGL·Ruxolitinib_ structures were conducted using the Desmond [47] in Schrodinger software. Orthorhombic periodic boundary conditions were utilized to define the size and shape of the repeating unit buffered at 10 × 10 × 10 Å distances. The system was solvated with water molecules adopting TIP3P model, electrically neutralized with sodium (or chloride) ions to balance the system charge, and finally 150 mM NaCl was added using OPLS2005 force field. The solvated system containing protein and ligand was minimized and relaxed for 100 ps via minimization step of Desmond using OPLS2005 force field. MD simulations were implemented with the periodic boundary conditions in the NPT (isothermal and isobaric simulation) ensemble. Martina-Tobias-Klein method [48] and Nose-Hoover thermostat algorithm [49] were used for isotropic pressure (1 atm) and constant-temperature (300 K), respectively. Total 100 ns NPT production simulations for DCLK1_KD·Ruxolitinib_ and JAK2_6VGL·Ruxolitinib_ structures were run and saved as trajectories at 100 ps intervals. The trajectories were analyzed using Simulation Interaction Diagram in Desmond.

### 4.8. Data Deposition

The coordinate and structure factor of the native DCLK1_KD·Ruxolitinib_ are available in the Protein Data Bank (https://doi.org/10.2210/pdb7F3G/pdb (accessed on 6 August 2021)) under accession code 7F3G.

## 5. Conclusions

Although DCLK1 is an emerging therapeutic target in gastrointestinal cancers, the development of an DCLK1 inhibitor has been proceeded only using compounds with the same scaffold. Through the screening of clinically applied compounds, we identified ruxolitinib as an inhibitor for DCLK1 with a novel scaffold. However, considering that ruxolitinib has many side effects such as thrombocytopenia, anemia, and neutropenia and shows a low binding affinity for DCLK1, the development of new inhibitors that are selective and potent for DCLK1 should be directed. Our structural analysis using the crystal structure of DCLK1 in complex with ruxolitinib suggests a possible approach for further design and optimization of selective and therapeutically effective inhibitors against DCLK1.

## Figures and Tables

**Table 1:** Data collection and refinement statistics for the DCLK1_KD·Ruxolitinib_ crystal structure.

PDB Entry	7F3G
*Data collection*	
Diffraction source	SPring-8 BL44XU
Wavelength (Å)	1.0000
Temperature (K)	100
Space group	*P*2_1_
a, b, c (Å)	57.248, 80.118, 59.338
α, β, γ (°)	90.000, 90.662, 90.000
Resolution range (Å) *^a^*	47.68–2.09 (2.21–2.09)
Total no. of reflections *^a^*	118,719 (17,485)
No. of unique reflections *^a^*	59,261 (9116)
Completeness (%) *^a^*	95.5 (90.8)
Redundancy *^a^*	2.0 (1.9)
〈I/σ(I)〉 *^a^*	8.41 (1.04)
*R*_sym_ *^a^*	0.050 (0.725)
*R*_meas_ *^a^*	0.067 (0.982)
*CC*_1/2_ *^a^*	0.998 (0.622)
*Model refinement*	
Overall B factor from Wilson plot (Å^2^)	44.0
No. of reflections, working set *^a^*	29,588 (2100)
No. of reflections, test set *^a^*	1568 (131)
final *R*_work_ *^b^*	0.210
final *R*_free_ *^b^*	0.253
No. of non-H atoms	4333
protein	4236
ruxolitinib	46
water	51
Average B factors (Å^2^)	67.04
protein	67.27
ruxolitinib	61.19
water	52.89
Root-mean-square deviations	
bonds (Å)	0.0048
angles (°)	1.1384
Ramachandran plot *^c^*	
favored (%)	97.94
allowed (%)	2.06
Rotamer *^c^*	
favored (%)	98.29
allowed (%)	1.71

*^a^* Values in parentheses refer to the highest resolution shell. *^b^* *R*_work_ = Σ||*F*_obs_| − |*F*_calc_||/Σ|*F*_obs_|, where *R*_free_ is calculated for a randomly chosen 5% of reflections, which were not used for structure refinement and *R*_work_ is calculated for the remaining reflections. *^c^* Values obtained using MolProbity.

## Data Availability

The data presented in this study are available in the article and Supplementary Materials files.

## Supplementary Materials

The following are available online at https://www.mdpi.com/article/10.3390/ijms22168488/s1.
