# Supplementary material for: Structural Basis of Inhibition of DCLK1 by Ruxolitinib
Source: Int J Mol Sci. 2021 Aug 6;22(16):8488. doi: 10.3390/ijms22168488 (PMC8395186; doi:10.3390/ijms22168488)
Supplement: Supplementary file 1 [file ijms-22-08488-s001.zip › ijms-1302819-supplementary.pdf]

International Journal of
Molecular Sciences

Article

Structural Basis of Inhibition of DCLK1 by Ruxolitinib

Dong Man Jang[1,†], Hyo Jin Lim[1,†], Hyunggu Hahn[1], Yeon Lee[1], Hark Kyun Kim[1,*] and Hyoun Sook Kim[1,*]

1 Research Institute, National Cancer Center, Goyang, Gyeonggi 10408, Republic of Korea; jdm721@ncc.re.kr (D.M.J.), mayhj4717@gmail.com (H.J.L.), hh2763@nyu.edu (H.H.), ylee@ncc.re.kr (Y.L.) hkim@ncc.re.kr (H.K.K.) and hskim@ncc.re.kr (H.S.K.)
* Correspondences: hkim@ncc.re.kr and hskim@ncc.re.kr; Tel.: +82-31-920-2275
† These authors contributed equally to the work.

Supplementary Data

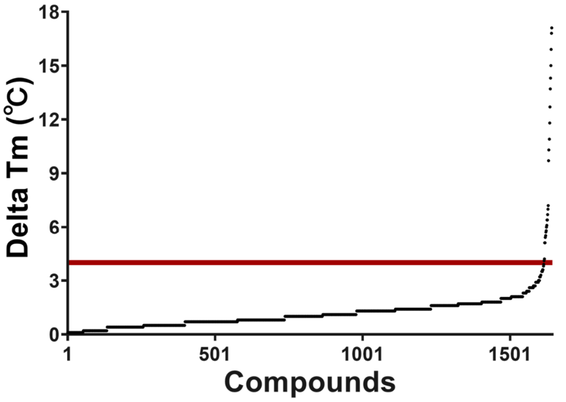

(**a**)

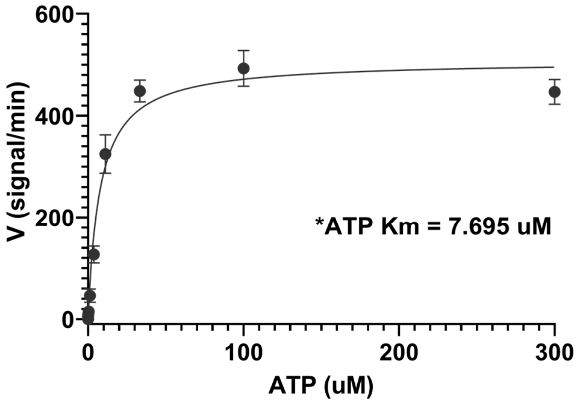

(**b**)

Figure S1. A clinical library screening against the purified DCLK1 kinase domain protein. (a) Delta melting temperatures of the DCLK1KD upon incubation of compounds against a control, DMSO, are plotted against the number of compounds from a library. Threshold for a significant Delta Tm value is set to the increased Tm shift of more than 4 °C indicated by red colored line. (b) Michaelis-Menten curve to determine Km of ATP for DCLK1 kinase domain. Using an HTRF assay, the velocity (signal/min) is measured against the concentration of ATP. All data are indicated as the mean ± SD of six independent experiments.

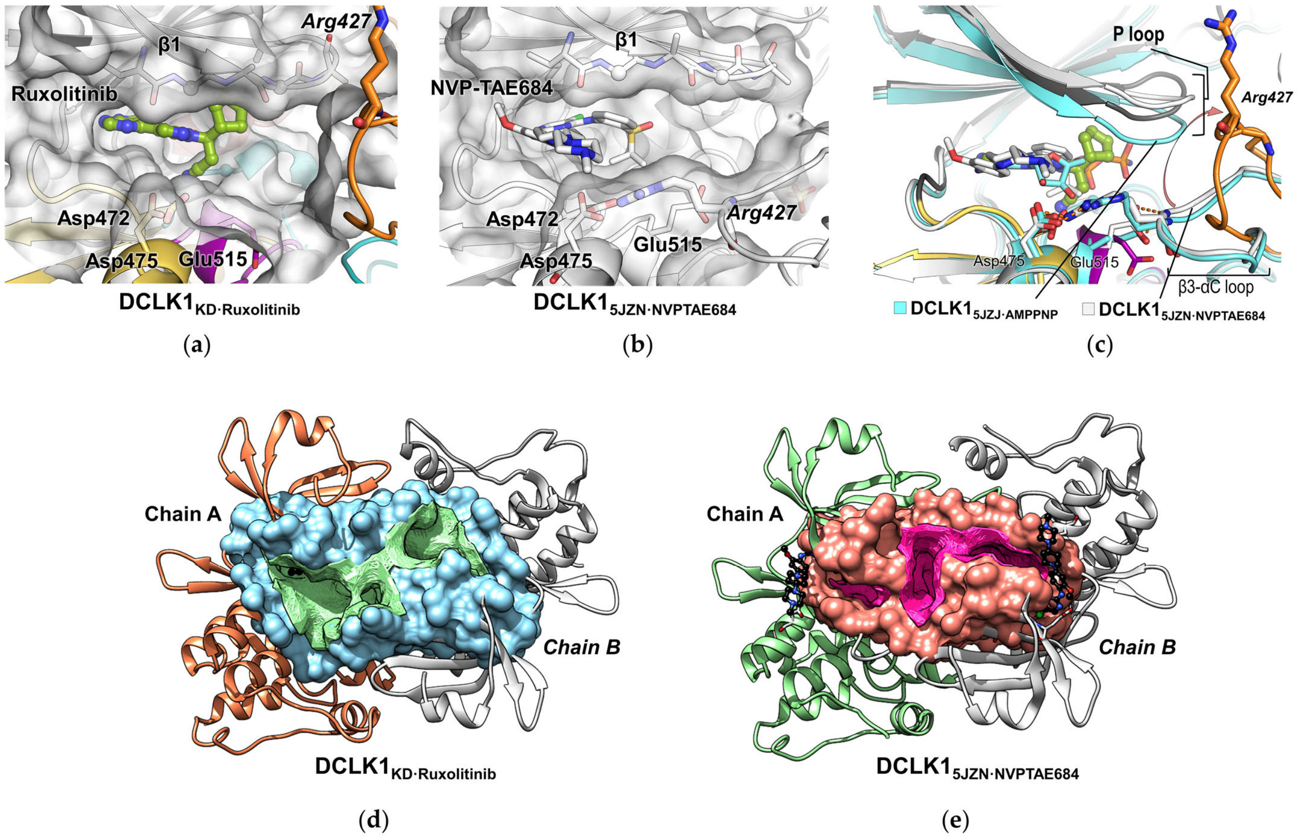

Figure S2. The active site and dimer interface of DCLK1$_{KD}$ upon the binding of inhibitors. (a, b) Binding pockets at the active site of (a) DCLK1$_{KD\cdot Ruxolitinib}$ and (b) DCLK1$_{5JZN\cdot NVPTAE684}$ (PDB ID: 5JZN) are shown as surface representations. (c) Superimposed view of the active sites of DCLK1$_{KD\cdot Ruxolitinib}$, DCLK1$_{5JZN\cdot NVPTAE684}$, and DCLK1$_{5JZJ\cdot AMPPNP}$ (PDB ID: 5JZJ). Inhibitors and key residues showing structural rearrangements are displayed as stick representations colored as indicated in the colored boxes. DCLK1$_{KD\cdot Ruxolitinib}$ is colored as in Figure 4b and Supplementary Figure S2a. (d, e) The overall dimer structures of (d) DCLK1$_{KD\cdot Ruxolitinib}$ and (e) DCLK1$_{5JZN\cdot NVPTAE684}$. The dimer interfaces of DCLK1$_{KD\cdot Ruxolitinib}$ and DCLK1$_{5JZN\cdot NVPTAE684}$ are shown with surface representations colored in blue and orange, respectively. Cavities formed from dimerization of DCLK1$_{KD\cdot Ruxolitinib}$ and DCLK1$_{5JZN\cdot NVPTAE684}$ are indicated as green- and pink-colored mesh representations, respectively.

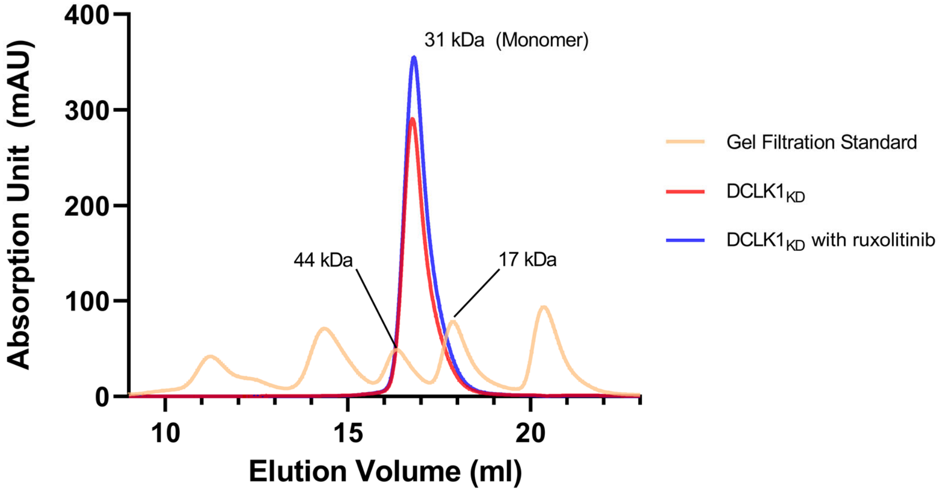

Figure S3. Oligomeric states of DCLK1KD upon ruxolitinib binding in solution. Oligomeric states of DCLK1KD (red) and DCLK1KD·Ruxolitinib (blue) were determined by size exclusion chromatography using Superdex 200 increase 10/300 GL (GE Healthcare, Chicago, IL, USA). Gel Filtration Stardard (light orange) (Bio-Rad #1511901, Hercules, CA, USA) was used to compare the molecular weight.

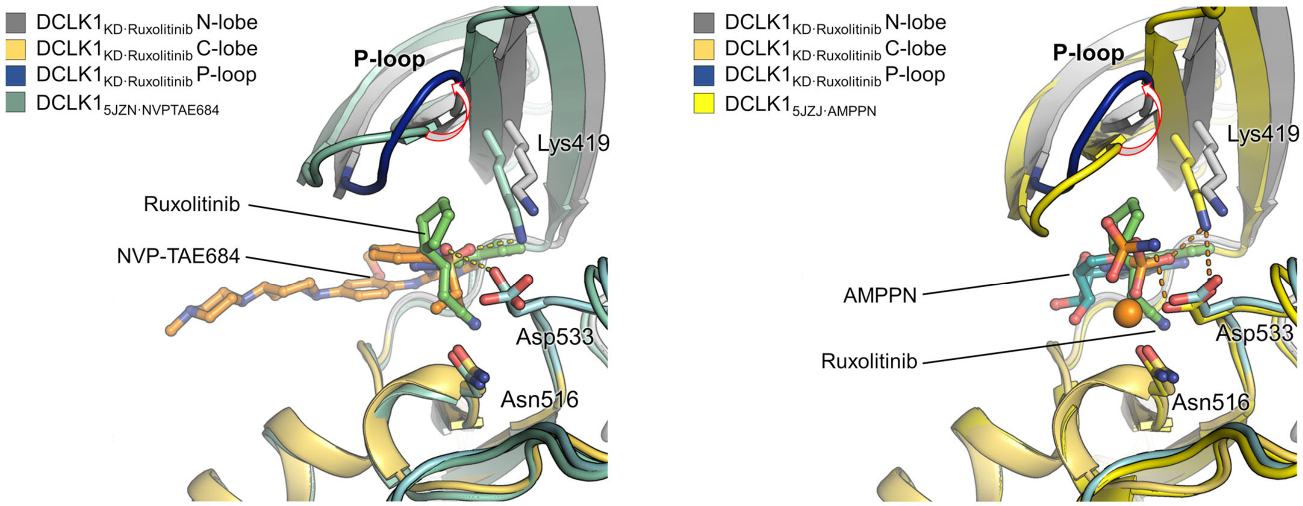

(**a**) (**b**)

Figure S4. The binding of inhibitors underneath the glycine-rich P-loop of DCLK1. (a, b) The structure of DCLK1$_{KD·Ruxolitinib}$ is superimposed to (a) DCLK1$_{5JZN·NVPTAE684}$ (green cyan) and (b) DCLK1$_{5JZJ·AMPPNP}$ (yellow). Inhibitors and the side chains of Lys419, Asn516, and Asp533 are shown as stick representations. All oxygen and nitrogen atoms are colored in red and blue, respectively. A magnesium ion is represented as a sphere colored in orange. Hydrogen bonds are indicated by red dotted lines.

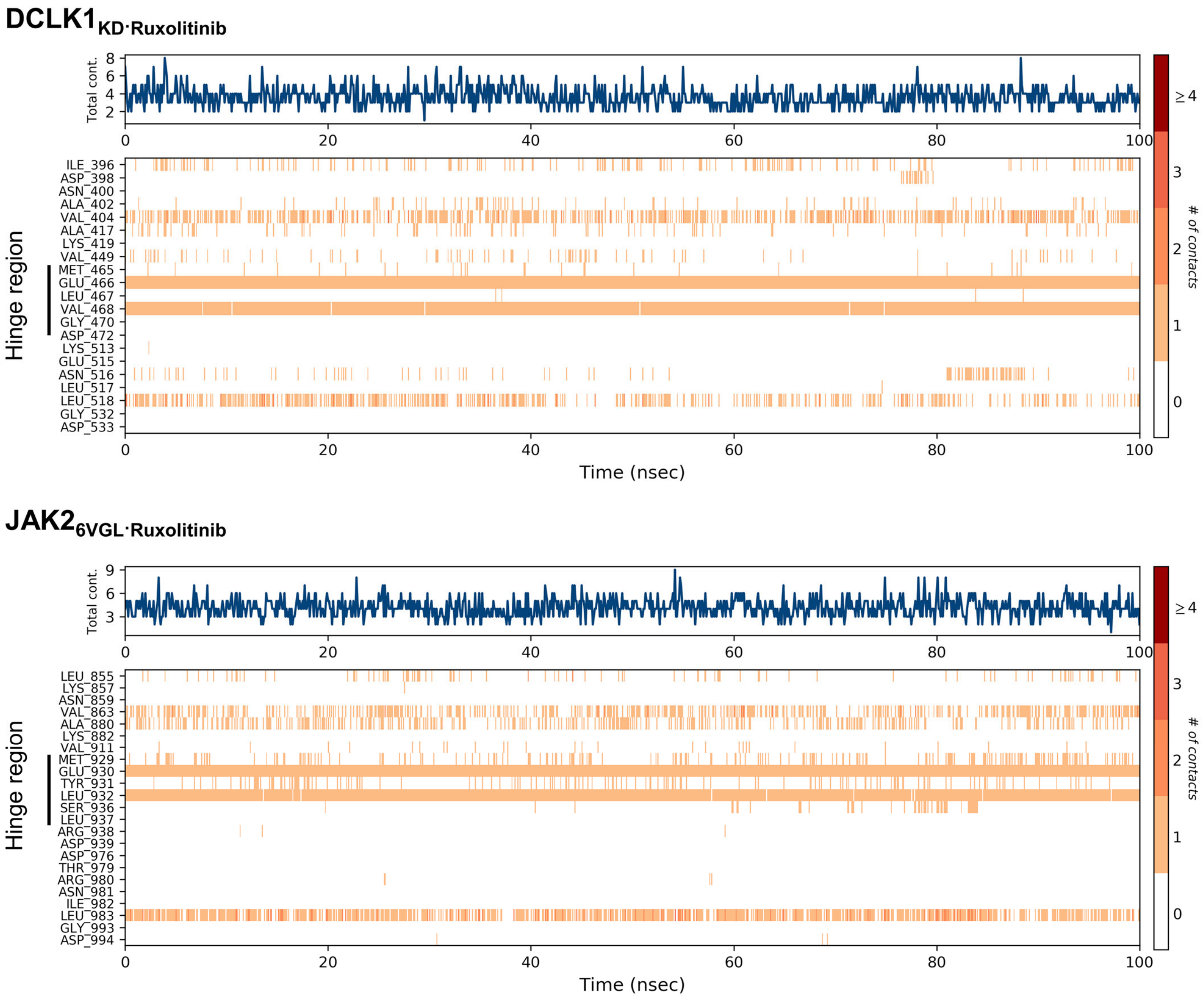

Figure S5. The number of contacts between DCLK1 (or JAK2) and ruxolitinib during 100 ns MD simulation. Total contacts between protein and ligand are indicated as blue lines in above boxes and contacts between residues of protein and ligand are indicated as orange lines in below boxes. The residues in the hinge region of DCLK1 or JAK2 kinase domains are displayed with black bold lines.

Supplementary Table S1. The 12 selected compounds from a clinical library screening.

ID	Chemical name	Chemical structure	Skeleton[a]
**1**	Zotarolimus (ABT-578)	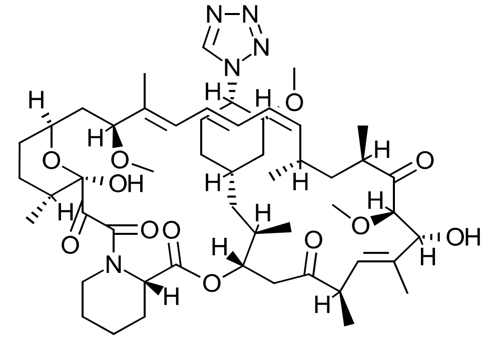	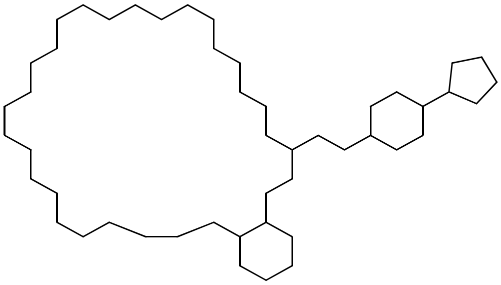
**2**	Cabazitaxel (Jevtana)	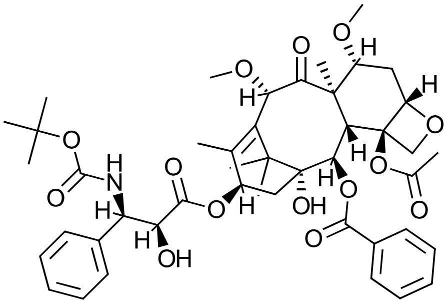	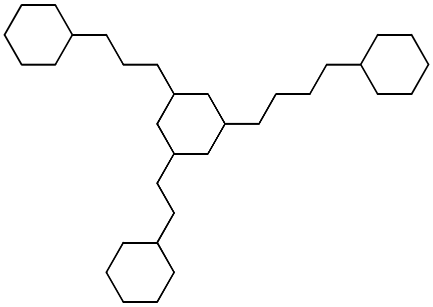
**3**	Sirolimus (rapamycin)	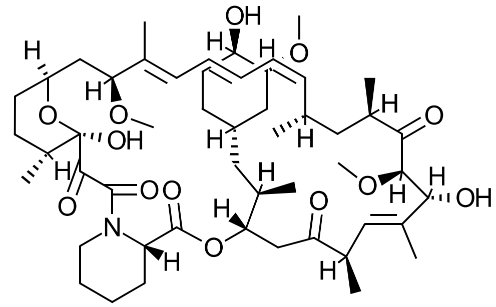	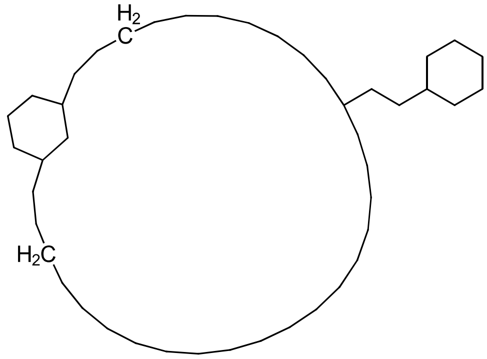
**4**	Clobetasol propionate	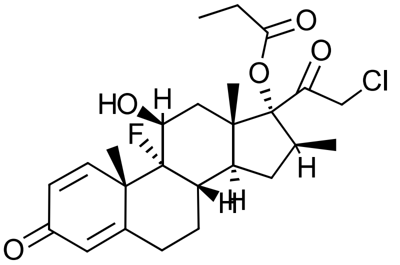	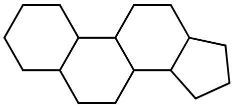

**5**	Vorapaxar (SCH 530348)	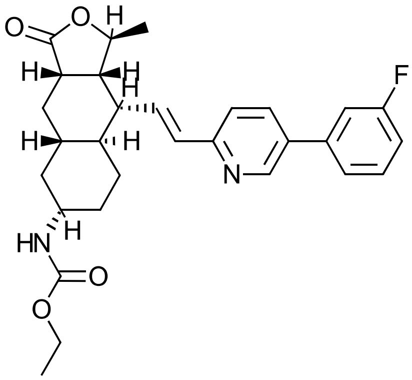	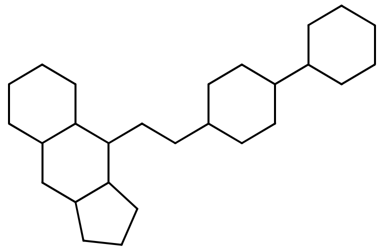
**6**	Ibipinabant (SLV319)	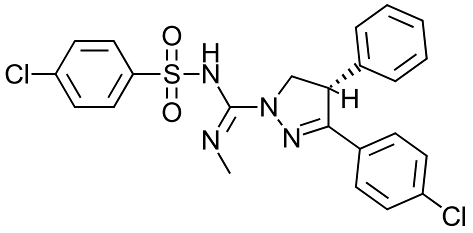	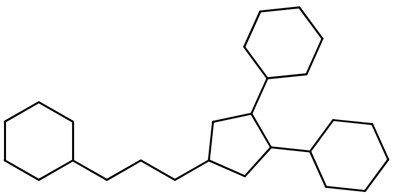
**7**	Diflorasone di-acetate (Psorcon)	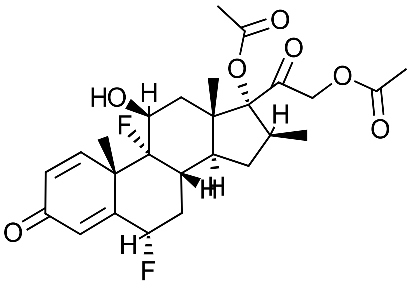	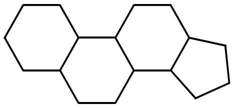
**8**	JTE-013	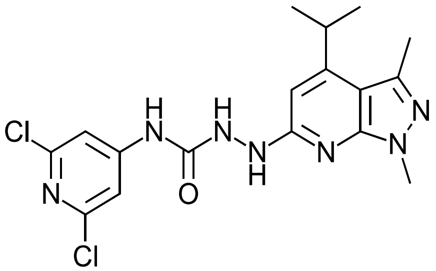	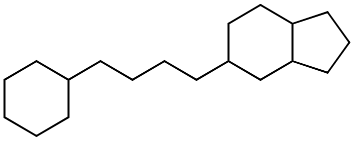
**9**	Ruxolitinib (INCB018424)	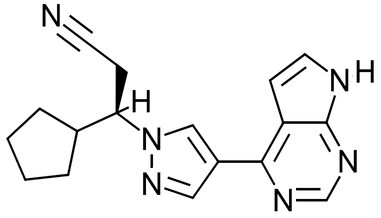	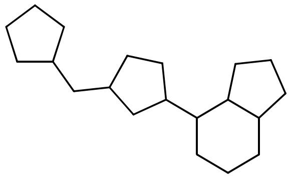

**10**	Azelnidipine	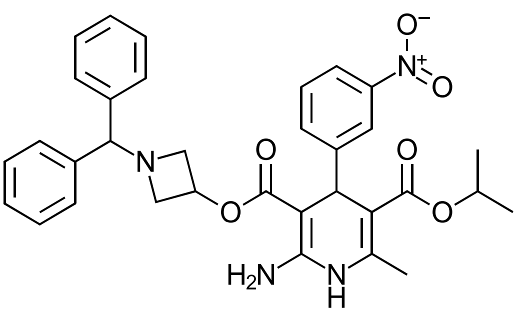	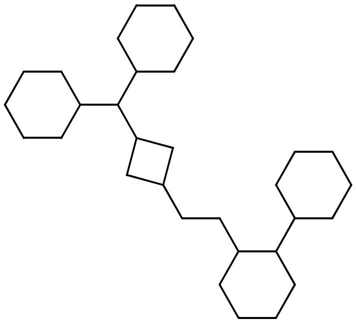
**11**	MDV3100 (Enzalutamide)	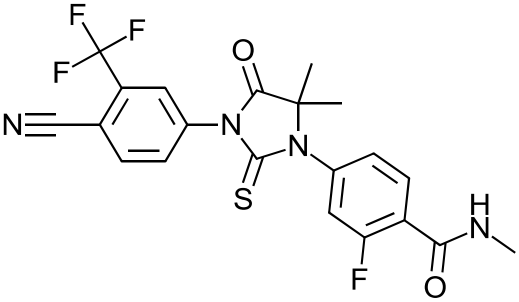	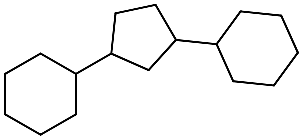
**12**	Manidipine (Manyper)	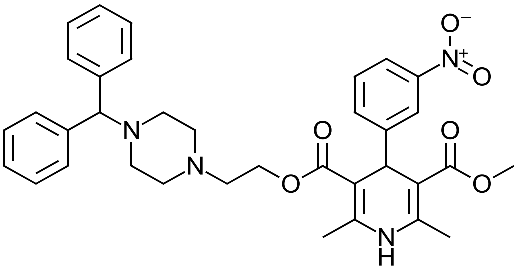	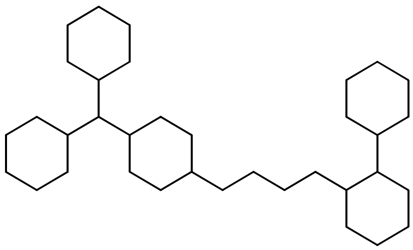

[a] Skeleton structures obtained using DataWarrior [1].

Reference

1. Sander, T.; Freyss, J.; von Korff, M.; Rufener, C. DataWarrior: an open-source program for chemistry aware data visualization and analysis. *J Chem Inf Model* **2015**, *55*, 460-473, doi:10.1021/ci500588j.
